# Acute morbidity profile and treatment seeking behaviour among people residing in an urban resettlement colony in Delhi, India 

**DOI:** 10.3126/nje.v8i1.21140

**Published:** 2018-03-31

**Authors:** Sarika Palepu, Kapil Yadav, Farhad Ahamed, Anil Kumar Goswami, Baridalyne Nongkynrih, Chandrakant S Pandav

**Affiliations:** 1 Senior resident, Department of community medicine and family medicine, All India Institute of Medical Sciences, Bhubaneswar; 2 Associate Professor, Centre for Community Medicine, All India Institute of Medical Sciences, New Delhi.; 3 Senior resident, Centre for Community Medicine, All India Institute of Medical Sciences, New Delhi.; 4 Associate Professor, Centre for Community Medicine, All India Institute of Medical Sciences, New Delhi.; 5 Professor, Centre for Community Medicine, All India Institute of Medical Sciences, New Delhi.; 6 Former Professor and Head, Centre for Community Medicine, All India Institute of Medical Sciences, New Delhi.

**Keywords:** Acute illness, morbidity, health care facility, utilization, India

## Abstract

**Background:**

Rapid urbanization has resulted in increased burden of communicable and non-communicable diseases, especially among urban poor population. In the absence of a well-functioning three tier health care system in urban India, health needs of urban poor are rarely fulfilled. The objective of this study was to assess primary health care services utilization pattern and its associated selected socio-demographic determinants in an urban population of Dakshinpuri Extension, South-east district of Delhi.

**Materials and Methods:**

A community based cross-sectional study was done from November 2013 to November 2014 with a sample size of 440 households through simple random sampling. Information was obtained regarding the socio-demographic characteristics and morbidity pattern of all the members of household in the preceding one year of the conduct of the present study through a pretested semi structured interview schedule. Association of various socio-demographic characteristics with primary and secondary health care facilities utilisation was studied with bivariate and multivariate logistic regression.

**Results:**

In this study, 42% of the household members suffered from acute illnesses and symptoms in the preceding one year. Secondary/tertiary health care facilities were approached mostly for seeking treatment. Majority of the household members sought treatment from private health care facilities. Significantly higher utilisation of secondary/tertiary health care facilities was found by head of households and household members who are married.

**Conclusion:**

Primary health care system needs to be revamped to improve healthcare delivery among urban population. Strategies to decongest secondary/tertiary health care facilities in urban India needs focus.

## Introduction

Urbanization is one of the leading global trends of the 21st century. It is expected that by 2050, about 70% of the world’s population will live in cities [1]. India has also undergone rapid urbanization with urban population increasing from 286 million (27.8% of total population) in 2001 to 377 million in 2011 (31.1% of total population) [2].

However the living conditions of urban population are jeopardised and have not grown on par with the increasing urbanisation. Globally, over a billion people reside in overcrowded and mortal situations in urban slums and unhygienic settlements [3]. Around 807 million city dwellers (one fourth globally) do not have access to improved sanitation. It is highly imperative to focus on reforming urban health because more than 170 million people defecate in the open space and 500 million people share sanitation facilities [4]. 

Also, the health statistics of urban dwellers in India vary widely in regard to the socio-economic status. Health indicators of urban poor are much below their urban and even rural counterparts. It was seen that among urban poor, only a quarter of pregnant females received complete antenatal care. Another alarming concern to focus in urban poor areas was that around 75% deliveries took place at home [2]. Amongst urban poor, about 59 per cent of women and 71.4 per cent of children suffer from anaemia. Around 47.1 per cent of under-five children were malnourished and 54.2 per cent were stunted. Enrolment of children was meagre among urban poor with only 53 per cent coverage under Integrated Child Development Scheme. Only 1 in every 10 women had regularly contacted a front line health care worker. This has a detrimental effect on the nutritional status of mother and child [5].

Urbanisation also leads to adverse health outcomes. It leads to increased susceptibility of suffering from both communicable and non-communicable diseases [6]. Evidence through literature reveals that urbanisation poses individuals at a greater risk of acquiring type 2 diabetes mellitus, hypertension, metabolic syndrome, [7,8,9] hearing impairment, sleep disturbances, stress disorders and cognitive impairments [10].

Provision of effective primary health care through a three tier referral system is lacking in urban areas. It was often assumed that mighty spread of health care facilities (with establishment of more private health care facilities) would address the health needs of the urban population. Currently, only 1083 urban family welfare centres and 871 health posts exist in the urban areas indicating the huge need for establishment of primary health centres [11]. A holistic approach with respect to health care utilisation pattern and their determinants should be made to address the health needs of urban, particularly urban poor population.

The objectives of the present study were to study primary health care services utilisation pattern and its association with selected socio-demographic determinants in urban population of Dakshinpuri Extension, South-east district, Delhi.

## Methodology

### Study design

This community based cross-sectional study was done in urban resettlement colony, Dakshinpuri Extension, Dr.Ambedkar Nagar, South-east district, Delhi. This area has been the field practice area of Centre for Community Medicine (CCM), All India Institute of Medical Sciences (AIIMS), New Delhi since 2002. 

### Sample size calculation

Taking prevalence of ever utilisation of primary health care services from similar settings as 45, [12], absolute precision as 5% and non-response rate as 10%, the estimated sample size was 440. In this study, 440 households were approached for studying the comprehensive utilisation of health care services by all the members of the household.

### Interview schedule design and validation

A semi structured, pretested interview schedule was prepared for the study. The domains of interview schedule consisted of socio demographic characteristics of all members of household, history of occurrence of acute illness episode or symptom in any member of the household, morbidity profiling, and health care provider/facility approached at first point of contact. Pre-testing of the interview schedule was done among 40 individuals (in a site different from the study area) and changes were made accordingly.

### Inclusion Criteria

Households residing in the study area for at least last six months and provided consent were included in the study.

### Exclusion criteria

People who were unable to comprehend interview questions were excluded from the study.

### Study respondent and participants

Head of the household was the study respondent. Information regarding all the members of the household was obtained from the head of the household.

### Data Collection

The study was conducted from November 2013 to November 2014. All the 19 blocks of the study area were visited apriori and information was obtained regarding the number of houses in each block. With the aid of the random number generator software, random number sequence was generated. Twenty three houses from each block (representing equal selection from every block as the sample size was 440 households) were selected by simple random sampling. Head of the household (based on the criteria of decision making) was the study respondent. If at the time of visit, head of the household was not available, the eldest female member of the family was selected as the study respondent. Information was obtained from the study respondent regarding the socio-demographic characteristics, morbidity profile of the preceding one year and health care facility approached at the first point of contact of all the members of household. In individuals who had two or more illness episodes or symptoms concurrently, the one which was perceived as more grave and/or which led to some therapeutic measure (either home remedy or visit to a health care facility) was considered. 

### Outcome variable

Primary outcome variable of the study was to assess the health care services utilisation pattern. Studying the association of health care services utilisation with selected socio-demographic variables was the planned secondary outcome variable.

### Explanatory variable

Acute illness episodes or symptoms in all the members of the household were assessed for explaining the primary and secondary health care services utilisation. For association of the health care services utilisation, selected socio-demographic variables were assessed.

### Ethical committee approval

Ethical clearance for the present study was obtained from the Institutional Ethics Committee, All India Institute of Medical Sciences, New Delhi with the reference number IESC/T – 38/03.01.2014 in January 2014..

### Data Management and statistical analysis

Data was entered in Epi Info 7.1 and was analyzed using STATA 12 version. For analysis, primary health care services were taken as any government primary health care facility, qualified private practitioner facility, Indian System of Medicine practitioner facility and informal health care practitioner (quacks) facility where in primary health care was provided. For analysis of secondary/tertiary health care services, both government and private sector secondary/tertiary health care facilities were included. Bi-variate analysis (with chi-square test) was done for studying the association between selected socio-demographic variables and health care services utilisation. Variables which were significant (with a p value of <0.2) on bivariate analysis were included for multi-variate logistic regression analysis. For determining significant association of selected socio-demographic variables with health care services utilisation in multi-variate logistic regression analysis, p value of <0.05 was taken.

## Results

A total of 440 households were visited for the present study. Among them, six study respondents refused to participate in the study (denied consent), 5 of them were residing in the study area for less than six months and 10 houses were locked despite two visits. A total of 419 were approached and head of the households interviewed. From these 419 households, information about 1801 individuals was obtained. The non-response rate of this study was 4.8%.

Majority of the male and female household members (55% and 56% respectively) belonged to the age group of 15-45 years. Median age of the members was 28 years (range - 4 months to 92 years). Among them, majority were married (49%), completed middle school (21.9%) with 9.5 mean years of schooling, males were either unemployed or students (30.4%) and females were home makers (71.1%) (Table-1). Majority of the head of households were males (83.0%) within age range of 40-60 years. Most of them completed high school (28.4%) and were skilled workers (38.5%). Female head of the households mostly were illiterate (64.8%) and were home makers (67.6%) (Table-2). The mean household size was 4.3 (range - 1 to 13). Majority of the households were nuclear by type (76%) and belonged to upper-lower category (41.3%) according to Kuppuswamy socio-economic status classification (13). 

Out of 1,801 household members, 760 had at least one acute episode of illness or symptom (range -1 to 6) in the preceding one year. A total of 1,050 illness episodes or symptoms were reported with 626 episodes in the past 3 months and 424 in the past 3-12 months. The incidence of morbidity per 100 individuals per year was 58.3 and per household per year was 2.5. Malaise was the most common symptom reported (19.5%) followed by respiratory tract infections (19.3%) and fever (15.0%) (Table-3).

Members of the household approached secondary/tertiary health care facility mostly (38.1%) for treatment of acute illness or symptoms. The other health facilities approached by them were informal health care practitioner (20%), qualified private practitioner (16.8%), government primary health care facility (14.7%) and Indian System of Medicine practitioner (5.7%). In 3.1% of illness episodes and symptoms, medications were sought from over the counter and in 1.6% of episodes, home remedies were used. (Table-4). In only 35% of acute illnesses or symptoms, Government health care facility (both primary and secondary/tertiary health care facility) was approached. Significant higher utilisation of secondary/tertiary health care facility services was seen among married household members and by head of the household. On multi-variate analysis, after adjusting for other variables, similar results were seen (Table-5). 

**Table 1:** Socio-demographic characteristics of the members of the household

Factors	Male	Female	Total
**Age group ( in years)**	**N (%)(n=921)**	**N (%)(n=880)**	**N (%)(n=1801)**
<5	83 (9.0)	56 (6.4)	139 (7.7)
6-14	124 (13.5)	124 (14.1)	248 (13.8)
15-45	506 (54.9)	496 (56.4)	1002 (55.6)
46-60	106 (11.5)	133 (15.1)	239 (13.3)
>60 (60-92)	102 (11.1)	71 (8.1)	173 (9.6)
**Marital Status**	**N (%)(n=921)**	**N (%)(n=880)**	**N (%)(n=1801)**
Married	448 (48.6)	435 (49.4)	883 (49.0)
Unmarried	441 (47.9)	368 (41.8)	809 (45.0)
Divorced/Separated	2 (0.2)	8 (0.9)	10 (0.5)
Widow/Widower	30 (3.3)	69 (7.8)	99 (5.5)
**Education**	**N (%)(n=828)**	**N (%)(n=815)**	N (%)(n=1643)*
Illiterate	103 (12.3)	205 (25.2)	308 (18.7)
Primary School Completed	124 (15.0)	129 (15.8)	253 (15.4)
Middle School Completed	190 (23.0)	170 (20.9)	360 (21.9)
High School Completed	183 (22.1)	117 (14.3)	300 (18.3)
Intermediate or Post-School Diploma	105 (12.7)	103 (12.6)	208 (12.7)
Graduation or PG completed	123 (14.9)	91 (11.2)	214 (13.0)
**Occupation**	**N (%)(n=658)**	**N (%) (n=647)**	N (%)(n=1305)**
Professional/Semi-professional	28 (4.3)	1 (0.2)	29 (2.2)
Clerk, Shop-keeper, Farmer	71 (10.8)	5 (0.8)	76 (5.8)
Skilled worker	185 (28.1)	32 (5.0)	217 (16.6)
Semi-skilled worker	87 (13.2)	17 (2.6)	104 (8.0)
Un-skilled worker	87 (13.2)	33 (5.1)	120 (9.2)
Unemployed/Student	200 (30.4)	99 (15.3)	299 (22.9)
Home-Maker	0	460 (71.1)	460 (35.2)

*Participants above the age of seven years were included **Participants above the age of 18 years were included

**Table 2:** Socio-demographic characteristics of head of households

Factors	Male(n=348)	Female(n=71)	Total (n=419)
**Age group ( in years)**	**N (%)**	**N (%)**	**Total N (%)**
22-40	134 (38.5)	11 (15.5)	145 (34.6)
40-60	137 (39.4)	29 (40.9)	166 (39.6)
>60	77 (22.1)	31 (43.7)	108 (25.8)
**Marital Status**	**N (%)**	**N (%)**	**N (%)**
Married	328 (94.3)	18 (25.4)	346 (82.6)
Unmarried	3 (0.9)	2 (2.8)	5 (1.2)
Separated/Divorced	1 (0.3)	2 (2.8)	3 (0.7)
Widow/Widower	16 (4.6)	49 (69.0)	65 (15.5)
**Education**	**N (%)**	**N (%)**	**N (%)**
Illiterate	71 (20.4)	46 (64.8)	117 (27.9)
Primary School	32 (9.2)	8 (11.2)	40 (9.5)
Middle School	74 (21.2)	8 (11.2)	82 (19.6)
High School Completed	99 (28.4)	6 (8.5)	105 (25.1)
Intermediate or Post-School Diploma	39 (11.2)	2 (2.8)	41 (9.8)
Graduation or PG completed	33 (9.5)	1 (1.4)	34 (8.1)
**Occupation**	**N (%)**	**N (%)**	**N (%)**
Professional/Semi-professional	27 (7.7)	0	27 (6.4)
Clerk, Shop-keeper, Farmer	50 (14.4)	0	50 (11.9)
Skilled worker	134 (38.5)	10 (14.1)	144 (34.4)
Semi-skilled worker	45 (12.9)	4 (5.6)	49 (11.7)
Un-skilled worker	56 (16.1)	9 (12.7)	65 (15.5)
Unemployed	36 (10.4)	0	36 (8.6)
Home-Maker	0	48 (67.6)	48 (11.4)

**Table 3:** Morbidity profile of acute illness episodes and symptoms among the members of the household

Type of acute illness episode	Total(n=1050)
Malaise	205 (19.5)
Respiratory tract infections	203 (19.3)
Febrile illness	158 (15.0)
Diarrhea	86 (8.2)
Skin infections	65 (6.2)
Injuries	45 (4.3)
Abdominal pain	39 (3.7)
Ophthalmological disorders	37 (3.5)
Gynaecological disorders	34 (3.2)
Others*	178 (17.0)

Others* - included ear infections (3.2), dental infections (2.9%), head-ache (2.8%), gastritis (2.5%), epistaxis (2.0%), palpitation (1.6%), vomiting (1.5%) etc.

**Table 4:** Distribution of acute illness episodes and symptoms by health care facility approached

Type of acute illness episode	Secondary/te rtiary care facility N (%)*	Informal health care practitioner N (%)*	Qualified private practitionerN (%)*	Government Primary health care facilityN (%)*	Indian System of Medicine practitioner N (%)*	Total (n=1000)
Malaise	72 (37)	43 (22.1)	23 (11.8)	41 (21)	16 (8.2)	195
Respiratory Infections	37 (19.5)	56 (29.5)	40 (21)	46 (24.2)	11 (5.8)	190
Fever	46 (29.7)	39 (25.2)	39 (25.2)	21 (13.5)	10 (6.5)	155
Diarrhea	18 (22.2)	28 (34.6)	22 (27.1)	11 (13.6)	2 (2.5)	81
Skin infections	34 (58.6)	11 (19)	6 (10.3)	5 (8.6)	2 (3.4)	58
Injuries	27 (61.4)	7 (15.9)	5 (11.4)	2 (4.5)	3 (6.8)	44
Abdominal pain	16 (43.2)	5 (13.5)	7 (18.9)	5 (13.5)	4 (10.8)	37
Ophthalmological disorders	27 (73)	1 (2.7)	5 (13.5)	3 (8.1)	1 (2.7)	37
Gynaecological disorders	13 (38.2)	4 (11.8)	8 (23.5)	7 (20.6)	2 (5.9)	34
Others	110 (65.1)	16 (9.5)	21 (12.4)	13 (7.7)	9 (5.3)	169

**Table 5:** Association of health care services utilisation pattern with selected socio demographic variables

Variable	Category	Health care services	Odds ratio (95% CI) [p-	Odds ratio(95% CI)
		Primary	Secondary	value] (Unadjusted)	[p-value] (Adjusted)
Age	15	288	193	1	-
	>16	312	207	1.1 (0.8-1.5) [0.4]	-
Sex	Female	351	231	1	-
	Male	249	169	1.0 (0.8-1.3) [0.8]	-
marital status	Unmarried/Divorced/ Separated	321	182	1	1
	Married	279	218	1.4 (1.1-1.8) [0.01]	1.4 (1.1-1.7) [0.03]
Education	Illiterate	206	135	1	-
	Literate	394	265	1.0 (0.8-1.4) [0.8]	-
Occupation	Unemployed	483	319	1	-
	Employed	117	81	1.0 (0.7-1.4) [0.8]	-
Type of family	Nuclear	420	284	1	-
	Extended	180	116	0.9 (0.7-1.3) [0.7]	-
Total family members	<4	283	189	1	-
	≥5	317	211	0.9 (0.8-1.2) [0.9]	-
Religion	Hindu	575	379	1	-
	Others	25	21	1.3 (0.7-2.4) [0.4]	-
Socio-economic status	Upper	325	211	1	-
	Lower	275	189	1.0 (0.8-1.4) [0.7]	-
Is person with illness, head of the household	No	480	293	1	1
	Yes	120	107	1.5 (1.0-2.0) [0.01]	1.4 (1.1-1.9) [0.03]

## Discussion

The present community based cross sectional study showed that 42% of the household members suffered from an acute illness episode or symptom in the preceding one year.

### Comparison of acute illness episodes

There were 1,050 acute illness episodes and symptoms among the members of the households. The study findings are consistent with prior research done in this arena. A study done in Tamil Nadu among 300 households also showed that 43% of participants had an illness episode in the past one year [14]. Human Development Survey-I conducted in rural and urban areas also revealed that 45% of the households suffered from an acute illness episode in the past one year [15]. 

In this study, malaise was the most common symptom reported (20%) followed by respiratory tract infections (19%) and fever (15%). Similar results were shown in few other studies [16,17,18,19,20]. 

### Comparison of health care facility utilisation

In the present study, in 35% of acute illness episodes or symptoms, government health care facility was approached and private health care facility was approached in the remaining 65%. According to Human Development Survey-I [18] and National Sample Survey Organization [21] also, in majority of the illness episodes (>70%), treatment was sought from private health care facilities.

Secondary/Tertiary health care facility was approached in most of the acute illness episodes or symptoms (38%) for seeking health care. Informal health care practitioners constituted an important source of curative health care provision for the households. A study done in Allahabad district, Uttar Pradesh also showed similar results wherein 32% participants sought treatment from non-registered practitioners, 2% from traditional care providers and 1% sought home remedies [22]. Another study done in West Bengal also revealed similar findings, where in treatment was sought from private allopathic practitioner in 11% episodes, informal health care practitioners in 4% episodes, ayurveda and homeopathic practitioner in 2% episodes, over the counter in 4% episodes and home remedies were sought in 14% of episodes [18]. 

The high response rate (95.2%) seen among the study participants along with simple random sampling methodology used ensured the representativeness in this study. Large sample size further strengthens the study findings. The socio-demographic characteristics of this study are similar to urban poor population as per Census 2011. Also, the present study findings corroborate with other studies done in similar settings. Hence, the results might be externally generalizable to the similar urban population.

### Recommendations

Predominance of private sector even in a marginalised population group like urban poor is a cause of concern. Concerted efforts to strengthen public sector health care, especially in primary health care should be made. Primary health care facilities should be strengthened to decongest secondary/tertiary health care facilities and also to provide health care facilities at the door-step of the needy. Effective referral mechanisms should be strengthened and community awareness should be generated for the same.Appropriate regulation and monitoring of health care providers should be done to limit the operations of informal health care practitioners. As an alternative, informal health care practitioners may be trained, accredited and mainstreamed with existing health care delivery system. Primary health care system also needs to be revamped and concerted efforts should be made for an effective public-private partnership in providing quality health care.

## Conclusion

There is a need to strengthen the primary health care system especially in the government sector to provide adequate health care to the urban poor. The present study showed that secondary/tertiary health care facilities were accessed as the main health care provider treatment of acute illness episodes or symptoms. Private sector health care providers, especially informal health care practitioners provided the bulk of curative health care services in the study area. 

### Limitation of the study

In the present study as all the illness episodes were self-reported, chances of potential measurement bias cannot be ruled out. Chance of recall bias is also possible in the study, as morbidity profile of preceding one year was enquired. The illness episodes of all family members were elicited from head of the household. This may lead to either under or over estimation of the prevalence.In this study, only the first point of contact health care facility was enquired. However, multiple health care facilities might have been visited for a single episode of illness. So, the study findings might not be representative of the overall health care seeking behaviour of the population. All the members of the household were included in the study. Modelling at the individual and household level was not taken into account for multi-variate logistic regression and hence multiple representations of the same household characteristics was possible in the analysis

### Future scope of the study

Research can be broadened in the field of urban health as it is presently a neglected paradigm. Focus can be extended to explore the various reasons for non utilisation of government primary health facilities for curative and preventive services. Through further research, an insight can be thrown into factors favouring the approach to informal health care practitioners for seeking treatment. Possible reasons for decongestion of secondary/tertiary health care services can be elicited through community interviews. Various contributing factors influencing the health care seeking behaviour can be studied on a larger scale and appropriate reformatory measures can be taken accordingly.

### What is already known on this topic?

Previous studies done in this area revealed that secondary/tertiary health care facilities were utilised mostly for seeking health care.

### What this study adds

This study analysed the morbidity profile of all members of the household over an extensive time period of one year. The three tier system of healthcare is not well utilised in the study area as majority of the household members visited secondary/tertiary health care facilities even for minor illnesses and symptoms treatment. Healthcare professionals/facilities approached at the first point of contact for all the acute illnesses and symptoms were elicited which captures the health care seeking behaviour of the community in total. This study also describes the widespread establishment of informal health care providers in the study area and urges a need for reform as most of them are untrained. This study establishes the need for strengthening the three tier referral system and quality health care provision.
